# Bilateral Chylothorax Due to Blunt Trauma Without Radiographic Evidence of Traumatic Injury

**DOI:** 10.5811/cpcem.2016.12.32937

**Published:** 2017-03-15

**Authors:** Mohamed Mohamed, Weaam Alshenawy, Christen Kegarise, David Betten

**Affiliations:** Sparrow Hospital, Department of Emergency Medicine, Lansing, Michigan

## Abstract

Chylothorax represents an uncommon clinical entity with multiple etiologies. Chylothorax following blunt thoracic trauma is typically associated with posterior rib fractures or thoracic vertebral fractures or dislocations. The occurrence of a traumatic chylothorax in the absence of associated injuries is a rare event. We report a case of a 51-year-old patient who developed bilateral chylothorax after sustaining blunt trauma without radiographic evidence of traumatic injury. A 51-year-old male presented to the emergency department complaining of progressively worsening shortness of breath and associated chest pain for the prior one week following a fall down several concrete steps. On evaluation, the patient was found to have bilateral pleural effusions with no radiographic evidence of traumatic injury, including posterior rib or thoracic vertebral fractures. Subsequent thoracentesis and pleural fluid analysis were performed confirming the diagnosis of chylothorax. Management included repeated thoracentesis, diet modification and octreotide administration, which resulted in dramatic improvement and eventual resolution of symptoms. Non-iatrogenic traumatic bilateral chylothorax in the absence of other radiographically demonstrated bony or soft tissue injury is a rare event. Chylothorax should be considered in the differential diagnosis of patients presenting with chest pain or shortness of breath following blunt trauma and evidence of pleural effusion, even in the absence of obvious traumatic fracture or injury on radiographic imaging.

## INTRODUCTION

Chylothorax is the accumulation of chyle in the pleural cavity and represents a rare clinical entity. It occurs due to disruption of the thoracic duct with etiologies that are primarily non-traumatic. Specifically, traumatic chylothorax is very uncommon with a reported incidence of 0.2–3.0% following blunt thoracic trauma and 0.9%–1.3% after penetrating trauma.^1^,^2^ Quincke was first to describe chylothorax in the setting of trauma in 1875 and its association with an ominous and grave prognosis. Mortality rates remain high with an incidence up to 15.5%.^3^ Diagnosis is often challenging and frequently delayed.^4^,^5^ The clinical scenario in the trauma setting typically involves associated rib or vertebral fractures, or dislocations that may directly disrupt the thoracic duct. The presence of significant bilateral chylothorax is an infrequently reported sequela of blunt trauma, especially in a patient without evidence of other injury. We herein report a case of a 51-year-old patient who developed bilateral chylothorax after sustaining blunt trauma in the absence of associated traumatic injury.

## CASE PRESENTATION

A 51-year-old male presented to the emergency department (ED) with progressively worsening shortness of breath associated with left-sided chest pain for one week. This followed falling down four concrete steps in which he had struck his back and chest against the staircase railing. While initially painful, symptoms improved over the next several days and the patient resumed his work as a manual laborer. By post-injury day five, there was acute and progressive worsening of symptoms, which led him to seek medical care. On presentation, the patient had chest pain of moderate intensity with radiation to the upper back, in addition to dyspnea worsened by coughing and supine position. He reported no fever, palpitations, syncope, dysphagia, nausea or vomiting. Past medical history was non-significant.

**Image 1(a–c):**
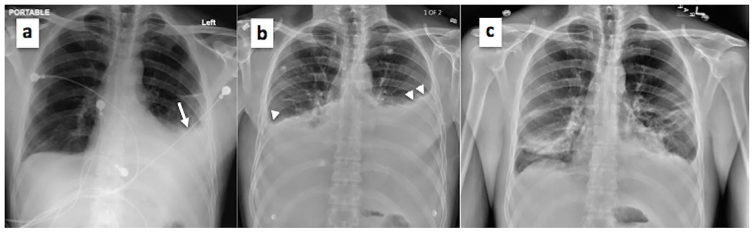
Plain radiograph of the chest showing (a) obliteration of the left costophrenic angle on initial presentation (white arrow); (b) reaccumulation of chylothorax with bilateral obliteration of costophrenic angles on second patient admission (white arrow heads); and (c) resolution of chylothorax effusion prior to final patient discharge.

**Image 2:**
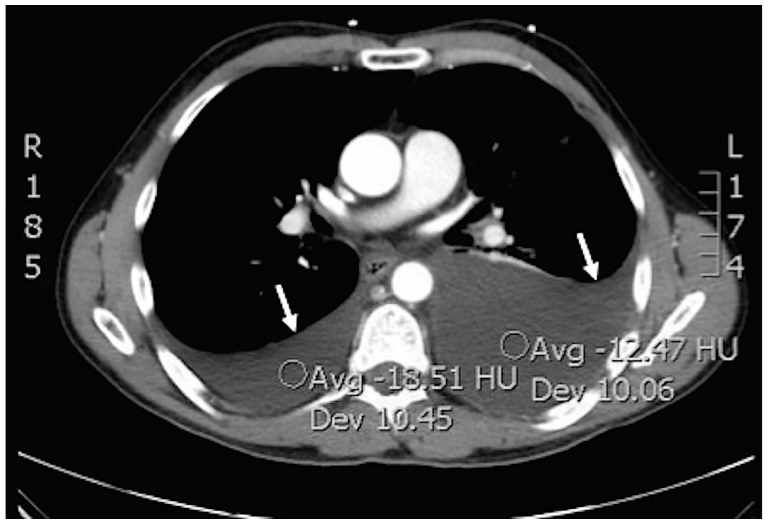
Computed tomography of the chest with contrast showing bilateral pleural effusions more remarkable on the left side (white arrows).

On examination, the patient appeared in obvious discomfort. Vital signs revealed a respiratory rate of 20 breaths/minute, heart rate of 65 beats/minute and blood pressure of 126/90, with an oxygen saturation of 92% on room air. Chest examination revealed diminished breath sounds on the middle and lower left lung zones. There was no chest wall tenderness, rhonchi or rales. Examination of other body systems was unremarkable. Plain chest radiograph demonstrated obliteration of the left costophrenic angle (Image 1a). Computed tomography (CT) of the chest with contrast revealed moderate to large left pleural effusion and small to moderate right pleural effusion with no evidence of pneumothorax, rib or vertebral fractures (Image 2). The patient was admitted to the hospital with bilateral pleural effusions of unclear etiology. A left-sided thoracentesis was subsequently performed and approximately 1.4 liters of milky white effusion were drained. Laboratory analysis of the pleural fluid was consistent with chylothorax revealing triglycerides of 4,750 mg/dL, leucocytes of 729 cells/uL, red blood cells of 1,000 cells/uL, LDH of 277 U/L, and total protein of 4.0 g/L, with absence of malignant cells on cytological analysis and no growth on microbiological examination.

The patient was placed on a fat-free diet with supplementation of medium chain triglycerides, monitored for worsening dyspnea, and assessed for fluid reaccumulation on serial radiographs. Upon improvement, he was discharged on post-admission day three while having only minimal dyspnea and with stable oxygen saturation. Three days later, however, his dyspnea worsened and he presented to the ED with oxygen saturation of 90% on room air and evidence of fluid reaccumulation on chest radiograph (Image 1b). Bilateral thoracentesis performed upon readmission drained two liters of chylous effusion from each hemithorax. Fat-free diet and medium chain triglycerides were reinstituted in addition to subcutaneous octreotide administration. Serial follow-up chest radiography remained stable (Image 1c) with no increase in effusion volumes, and the patient was subsequently discharged three days later. On follow up three weeks later, the patient was doing well.

## DISCUSSION

Chyle is composed of ingested fat from the gastrointestinal tract and lymphatic fluid from the peritoneal cavity and lower extremities.^2^ The thoracic duct represents the main vessel that transports chyle.^2^ Causes of non-traumatic chylothorax primarily include neoplastic, congenital, inflammatory, and idiopathic etiologies.^3^,^6^ Malignancy (usually lymphoma) is considered the most common cause in non-traumatic cases.^4^,^6^ In the less commonly occurring traumatic chylothorax, iatrogenic etiologies (approximately 80% of cases) are usually the cause.^2^,^7^ Because the thoracic duct is protected posteriorly by the thoracic and lumbar spine and anteriorly by the mediastinum, the occurrence of chylothorax after blunt trauma is uncommon.^5^,^7^ Classically, high velocity and/or sudden deceleration mechanisms are involved and the presence of nearby vertebral or posterior rib fractures, or vertebral dislocations is what usually disrupts the thoracic duct.^3^,^7^,^8^ Other mechanisms have been proposed to explain thoracic duct injury in the absence of fractures or dislocations. In a case by Apostolakis et. al., existing osteophytes/exostosis were suggested as being the likely culprit that resulted in puncturing of the thoracic duct following blunt trauma.^7^ Alternatively, after blunt trauma, sudden flexion/hyperextension of the lower thoracic spine or exposure to the shearing forces of the of the diaphragm may result in duct injury.^3^,^7^

We have encountered only a few case reports in the literature of isolated traumatic chylothorax in the absence of obvious significant traumatic injury. In one report, there were no chest or spine fractures; however, effusion was unilateral.^9^ In the second report, effusion was also unilateral but the involvement of associated fractures or dislocations on imaging was not documented, making it difficult to conclude that such injuries were non-existent, especially in the context of trauma being caused by a horn injury from a bull.^1^ In the remaining three reports,^3^,^5^,^7^ chylothorax was bilateral. Of those, there was similarly no documentation of fractures or dislocations in two reports;^3^,^5^ however, the mechanisms of injury were motor vehicle crash with bilateral hemothorax in one case, and water skiing injury at 40 mph in the other. Lastly was the case (mentioned earlier) reported by Apostolakis et. al.^7^ As such, it is likely that our case represents the fourth to be reported in the English-language medical literature with bilateral traumatic chylothorax in absence of obvious traumatic injury over the last decade.

Chylothorax typically possesses a latency period of 2–7 days from injury to the development of signs and symptoms, and a median of seven days from trauma to diagnosis.^5^,^9^ Two suggested possible reasons exist for this: 1) slow collection of chyle and lymph in the posterior mediastinum before rupturing into the pleural space up to 7 – 10 days later; and 2) possible relative interruption of normal diet following trauma; it is known that a fasting state may significantly reduce the chyle flow rate from the normal 100 ml/h after eating to 14 ml/h.^2^,^4^,^9^ This gradual evolvement makes chylothorax better tolerated and may delay symptoms and diagnosis until the effusion becomes large enough.

The clinical picture primarily consists of progressive dyspnea and chest pain. Electrolyte abnormalities may develop including hypocalcemia, hyponatremia and acidosis from continued loss of chyle into the pleural space.^2^,^4^,^9^ If left untreated, signs and symptoms of malnutrition and hypovolemia may prevail.^9^ Additional loss of immunoglobulins and T-lymphocytes can further lead to immunosuppression.^4^,^9^ Early diagnosis and recognition is warranted to avoid the detrimental effects of malnutrition, hypovolemia and immunocompromised state.

In the trauma setting, a plain radiograph demonstrating a pleural effusion often leads to subsequent CT to evaluate the possible underlying etiologies. Thoracentesis will demonstrate pleural fluid that is odorless, milky-white (in 50% of cases) but may be serous or serosanguinous if hemothorax is associated.^2^ Presumptive diagnosis is made via quantitative analysis of the pleural fluid that reveals a triglyceride level >110 mg/dl (99% diagnostic).^4^,^10^ Lipoprotein electrophoresis of the pleural fluid demonstrating chylomicrons is confirmatory and remains the gold standard, especially when triglyceride levels are below 110 mg/dl; however, it may not be readily available.^2^,^4^,^6^ In our case, this was not required because triglyceride levels were considerably higher. Fasting and nutritional status however, should be considered while interpreting results as it may contribute to lower triglyceride levels. Maldonado et, al. reported 14% of patients with chylomicron-positive pleural fluid but with triglyceride levels <100 mg/dl.^6^ Other secondary diagnostic characteristics of the pleural fluid in chylothorax may include a pH of 7.4–7.8, specific gravity of 1.012 or higher, lymphocytic predominance of >1,000 cells/ul, fluid to serum cholesterol ratio of <1, and a triglyceride ratio of >1.^6^,^9^,^10^ Lymphangiography may further demonstrate the site of injury.^6^ Pseudochylothorax, a cholesterol-rich effusion commonly associated with chronic inflammatory disorders, and empyema may also result in milky effusions. Differentiation may be achieved via the simple addition of ethyl ether to the fluid demonstrating disappearance of the milky appearance in the former, and fluid centrifugation displaying a clear supernatant in the latter.^2^,^4^

Conservative management consists of thoracentesis (to allow lung re-expansion), reducing chyle flow via starvation diet (to allow healing of the thoracic duct), and total parentral nutrition (TPN) to address nutritional and metabolic complications. Success rate of this approach is said to range from 20–80%.^2^ Using medium chain triglycerides is advocated because they are directly absorbed into the portal system bypassing the intestinal lymph system.^6^ Agents such as somatostatin and octreotide may also be used to reduce intestinal chyle production, thereby reducing chyle flow through the thoracic duct. Although in our case TPN was not instituted, the combination of thoracentesis, fat-free diet, medium chain triglycerides, and octreotide eventually led to the cessation of chylous effusion.

Patients who fail conservative management (usually within two weeks) with progression to malnutrition or electrolyte abnormalities may be candidates for surgical intervention.^2^ Timing, however, is individualized, with early surgical consultation recommended in all cases of traumatic chylothorax. Previously used criteria suggesting a need for surgical intervention include the following: 1) a chyle leak of >1.5 L/day; 2) output greater than 1L/day for five or more days; 3) persistent flow for > 2 weeks; or 4) rapid deterioration in nutritional status.^2^ Percutaneous thoracic duct embolization is a safe alternative to surgical intervention and represents the first line of treatment at some institutions with reported success rates of approximately 70%.^5^,^11^ However, despite having a lower morbidity rate compared to surgical intervention, it is performed in only a few centers, the reason possibly being the rarity of expertise in a technically challenging procedure.^5^,^11^

Overall, mortality rates have been lowered over the years due to introduction of various aggressive therapeutic approaches that aid in reversing the detrimental effects of chyle loss.^6^ Early recognition and diagnosis are still paramount for achieving better outcomes.

## CONCLUSION

Non-iatrogenic traumatic bilateral chylothorax is unusual, and its occurrence in the absence of posterior rib or thoracic vertebral fractures, as in our case, is exceedingly rare. Chylothorax should be considered in the differential diagnosis of patients presenting with chest pain or shortness of breath within one to two weeks following blunt trauma, even in the absence of traumatic fracture or injury on radiographic imaging. In our case, admission and conservative management led to excellent results.
